# Cutting Characteristics for Sugar Maple Using Single Grit with Spherical Cone and Triangular Pyramid Geometries

**DOI:** 10.3390/ma12213621

**Published:** 2019-11-04

**Authors:** Jian Zhang, Bin Luo, Li Li, Hongguang Liu

**Affiliations:** 1 College of Materials Science and Technology, Beijing Forestry University, No.35 Tsinghua East Rd, Haidian District, Beijing 100083, China; 2 MOE Key Laboratory of Wooden Materials Science and Application, College of Materials Science and Technology, Beijing Forestry University, No.35 Tsinghua East Rd, Haidian District, Beijing 100083, China

**Keywords:** wood sanding, single-grit cutting, grit geometry, material removal, energy consumption

## Abstract

Abrasive belt sanding plays an important role in wood processing. The abrasive grits on the belt perform similar to small cutting tools with negative rake angles. In this study, a series of single-grit scratching tests were carried out on Sugar maple workpieces to investigate the cutting characteristics of two different abrasive-grit shapes. The spherical cone grits had two kinds of included angles, and the triangular pyramid grits provided two cutting forms: one main cutting edge and two side cutting edges as well as two main cutting edges. Both scratching along and across the wood grain direction were conducted. In all cases, the material deformation and surface creation were analyzed, as well as cutting force. Several physical cutting models were established to help further understand the cutting mechanism. A new method was proposed to evaluate the energy consumption of single-grit scratching. The results showed that triangular pyramid grits with sharp cutting edges could sever wood fibers more efficiently, while spherical cone grits are prone to make material plastic deformation mainly manifested as superficial densification and pile-up. When scratching along the wood grain, the triangular pyramid grit with two main cutting edges showed the best cutting performance with better surface quality. It was also shown that the cutting force ratio of spherical cone grits was apparently less than that of triangular pyramid grits. The overall cutting power for spherical cone grits was remarkably higher than that for triangular pyramid grits for both scratching along and across the wood grain, which indicates that triangular pyramid grits have higher cutting efficiency and power utilization.

## 1. Introduction

Sanding is a commonly used method in wood machining, which mainly aims to remove a certain quantity of material and acquire a better surface quality prior to gluing and painting [1,2]. Abrasive grits on the sanding belt work as small cutting tools with low or even negative rake angles, which induce high normal forces on the wood surface [3]. At present, the literature of wood sanding is mainly about sanding forces, surface roughness, and the influence of surface quality on coating performance. However, there are a few published works dedicated to material removal and cutting efficiency in wood sanding.

Based on the grit-workpiece interaction, Hahn [4] divided the material deformation into three phases which are rubbing, ploughing, and cutting. Rubbing accompanies material elastic deformation. Ploughing is initiated with an increasing grit penetration, which primarily leads to material plastic deformation and consumes energy without contributing much to material removal [5]. When the shearing strength reaches a critical stage, part of the deformed material is completely torn out, and debris is removed during grit cutting. This model was established for metal grinding, and it has been referenced in wood sanding research for a long time. In fact, the material removal in sanding processes is characterized by a multitude of irregularly shaped, randomly arranged abrasive grits, which interact with the workpiece [6]. Zhang et al. [7] conducted a series of single-grit scratching tests aimed at investigating the material removal and surface creation for wood materials. In the research, elastic recovery at the rubbing stage was first verified and anatomical cavities’ absorbing effect was proposed to account for the tiny plastic pile-up at two sides of furrows.

The shape of grits exerts great influence on cutting characteristics. For instance, Xie and Williams [8] found that an increasing negative rake angle incurred higher material plastic deformation. Lee et al. [9] classified the grit shapes into four types: conical shape, spherical shape, circular table shape, and rectangular pyramid. Basically, the conical grit model has been widely used in finite element simulation. Anderson et al. [10] compared spherical and truncated cone geometries in single abrasive-grit cutting, using experimental observations and a validated finite element. Zhang et al. [11] did both experimental and numerical research on the single-grit scratching process of oxygen-free copper (OFC). A Rockwell indenter made of a natural diamond was selected as the tool and the corresponding 3D model was established. For grits with discernible cutting edges, Axinte et al. [12] investigated the impacts of the micro-cutting edges’ geometry on grinding. Three different shapes of grits (circular, square, and triangular) were manufactured by laser ablation and mounted on a dummy wheel. Yu et al. [13] investigated the grinding characteristics of optical glass BK7 experimentally. The single-grit tool was made by a single diamond grit with a triangular pyramid geometry. Similarly, Mei et al. [14] assumed that the initial geometry of CBN single grit was a rhombus with a very small plane on the top and bottom, and it numerically investigated the evolution of grit fracture and its impact on cutting performance. 

However, only grit size was considered a primary tool parameter in previous research on wood sanding. Moura and Hernández [3] took grit size and feed speed as sanding parameters to see how they influenced the sanded surfaces of Sugar maple wood. It was found that the superficial cell damage was reduced when the final grit size increased. Luo et al. [15] completed research on the effect of sanding parameters including grit size on sanding forces for a wood-based panel. The quantity of grits in the unit area becomes larger with bigger granularity. Generally, the sanding forces present a linear increase when granularity exceeds P80 for both a particle board and a medium-density fiberboard. However, very few works can be found referring to the effects of different grit geometries in wood sanding.

Wood is a kind of heterogenous material with an affluent porosity and fiber structure [16,17]. To further understand the material deformation and removal for wood sanding under different cutting modes, a series of single-grit scratching tests were designed and conducted on Sugar maple, using two types of grit shape (spherical cone and triangular pyramid). The cutting characteristics were determined and evaluated by surface morphology and cutting force analysis. The results present how the grit geometry affect material deformation and the corresponding energy utilization, which can provide an insight to develop a new type of abrasive tool used in wood sanding with better cutting efficiency and surface quality. 

## 2. Materials and Methods

### 2.1. Experimental Setup

**Figure 1:**
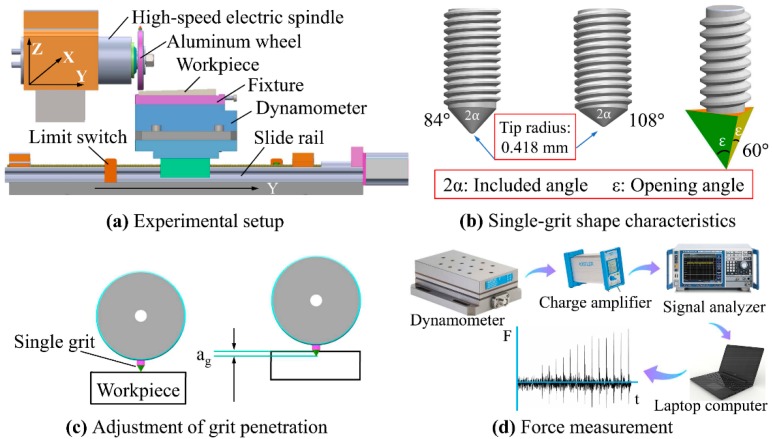
The single-grit scratch testing system: (**a**) experimental setup; (**b**) single-grit shape characteristics; (**c**) adjustment of grit penetration, and (**d**) force measurement.

To generate successive scratches on workpieces, a single-grit scratch testing system was designed and manufactured (see Figure 1a). The dummy wheel, driven by a precise electric spindle with 0.01 mm accuracy, was made from aluminum. The diameter of the aluminum wheel was measured as 100 mm and the rotational speed was set to be 2700 rpm. Two geometries were chosen to approximate the abrasive grits, as illustrated in Figure 1b (spherical cone tool and triangular pyramid tool). To study the effect of different negative rake angles on single-grit cutting, the included angles of two spherical cone tools were 84° and 108°, respectively. The triangular pyramid tool consisted of an opening angle of 60°. All the single-grit tools were made from 304 stainless steel and screwed to the aluminum wheel.

Before creating a new series of scratches, the wheel needs to be carefully adjusted so that the grit-workpiece contact is under a critical condition. Then the wheel moves downward in the Z-axis with an initial grit penetration depth a_g_ to conduct single-grit experiments (see Figure 1c). The normal and tangential forces are measured by using the system (see Figure 1d), which contains a three-component force dynamometer (KISTLER 3257A, Kistler Instrumente AG, Winterthur, Switzerland), a charge amplifier (KISTLER 5806, Kistler Instrumente AG, Winterthur, Switzerland), a signal analyzer (NEC Omniace II RA2300, NEC Corporation, Tokyo, Japan), and a PC (Y400, Lenovo Group Ltd., Beijing, China).

The electric spindle can move in a horizontal direction on the X-axis and a vertical direction on the Z-axis. The workpiece and dynamometer were mounted on the workbench through a special fixture. To clearly recognize the scratches, the feed rate of the workpiece was set at 9 m/min considering the rotational speed of the wheel. One side of the workpiece was inserted with a metallic gasket so that scratches were created with different cutting depths a_gmax_ (0–0.5 mm). When the workpiece reaches a certain point along the Y-axis, the limit switch will be triggered to lift the grit up on the Z-axis.

### 2.2. Workpiece Preparation

Sugar maple (*Acer saccharum*), which is a diffuse-porous hardwood commonly used for indoor applications, was selected in this study. Prior to workpiece preparation, the Sugar maple boards with no clear defects were levelled with 80 grit aluminum oxide abrasive belt and then finished with a 120 grit aluminum oxide abrasive belt (SANDTEQ W-200, HOMAG, Schopfloch, Germany) to produce initial flat surfaces with low roughness. Then the boards were cut at dimensions of 130 mm (Length) × 30 mm (Width) × 30 mm (Thickness). The average density of all workpieces was 0.83 ± 0.02 g/cm^3^. To avoid the effects of moisture content, all the workpieces were conditioned at 65% ± 5% relative humidity (RH) and 20 ± 1 °C for a week to reach an equilibrium moisture content of approximately 11.2%.

### 2.3. Cutting Direction Definition

**Figure 2:**
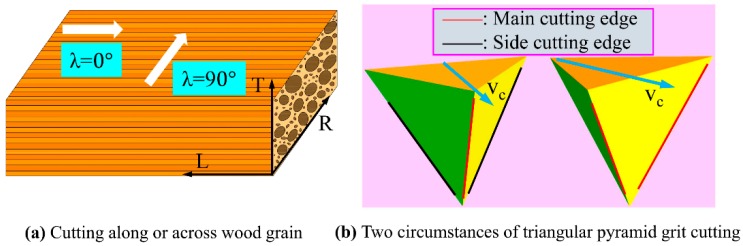
Schematic diagram of a single-grit cutting direction: (**a**) cutting along or across wood grain, and (**b**) two circumstances of triangular pyramid grit cutting.

Orientation of wood fibers greatly influence mechanical behavior such as material deformation and cutting forces. In this study, two typical cases of cutting along (λ = 0°) and across (λ = 90°) wood grain were performed, as demonstrated in Figure 2a, where λ refers to the angle between the directions of grit movement and wood grain. Since the main cutting edge and side cutting edge play different roles in the rupture of wood fibers, two circumstances were designed for the triangular pyramid grit for both λ = 0° and λ = 90°. Specifically, the first circumstance presents a main cutting edge and two side cutting edges, and the other circumstance presents two main cutting edges, as shown in Figure 2b.

### 2.4. Scratch Profile Measurement and Topography Observation

The scratch profiles on the tested workpieces were measured by a 3D profilometer (KEYENCE VR-5000, KEYENCE, Osaka, Japan), with a measuring resolution of 0.5 μm on the Z-axis. To minimize the influence of air humidity variation, a series of scratches on each workpiece were scanned as soon as the scratches were generated. Using analyzing software (VR Series version 3.2.0.67), 2D cross-sectional profiles were extracted from the deepest point of scratches. The scanning electron microscope (SEM) observations were conducted for representative scratches by using scanning electron microscopy (JSM-6700F, JEOL, Tokyo, Japan).

### 2.5. Cutting Force Ratio and Cutting Power Calculation

One method to evaluate the cutting efficiency of single grit is to calculate the overall specific energy *S*, which is applied to measure the energy required to displace a unit volume of material. It is calculated as [18]:

(1) $$S\;=\;\frac{E}{V}$$

where *E* is simply the consumed power of the scratching process and *V* is the volume of the groove cut by single tools.

Wood is a porous material. The elastic spring-back effect will affect the precision of the measurement of *V* using a 3D profilometer. Therefore, the cutting force ratio $F_{t}$/$F_{n}$ was used in this study to evaluate the cutting performance of single grits.

**Figure 3:**
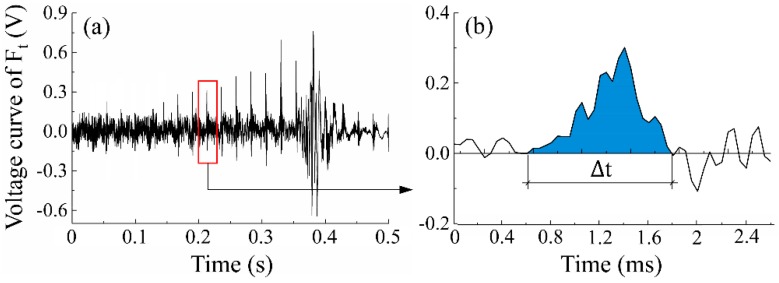
The integration processing of the voltage curve of tangential force *F_t_*: (**a**) a typical voltage curve of *F_t_* covering several scratches, and (**b**) an extracted voltage curve of *F_t_* for a single scratch.

Meanwhile, overall cutting power *P* was calculated to indicate the difference in energy consumption for single grits, which is shown in the equation below.

(2) $$P=\frac{W}{\Delta t}=\frac{\int_{t_{1}}^{t_{2}}F_{t}\left(t\right)v_{c}dt}{t_{2}-t_{1}}=\frac{v_{c}k\int_{t_{1}}^{t_{2}}U\left(t\right)dt}{t_{2}-t_{1}}$$

where *W* is the cutting power of tangential cutting force $F_{t}$ during the scratching process, which can be calculated in Origin 9.1 Pro (OriginLab Corporation, Northampton, UK) by using the integrating function based on the force curve (see Figure 3). *U* is the collected voltage value of the force signal, and *k* is a conversion coefficient that can be calibrated prior to the tests [15].

Since the grit-workpiece interaction time is very short with several microseconds and the penetration depth is relatively small, the velocity of the cutting edge can be regarded as equal to the instant cutting speed $v_{c}$, which is shown in the equation below.

(3) $$v_{c}=\frac{\pi Dn}{60}$$

## 3. Results and Discussions

### 3.1. Surface Creation and Cutting Mechanism

One of the goals of this study is to figure out the material deformation and removal process for cutting tools with varied geometries. For wood materials, scratching direction exerts great influence on fiber rupture and chip formation. From this point of view, the surface topography and theoretical cutting models of scratching along and across wood grain were, respectively, analyzed and established in this part.

#### 3.1.1. Scratching along the Wood Grain (λ = 0°)

**Figure 4:**
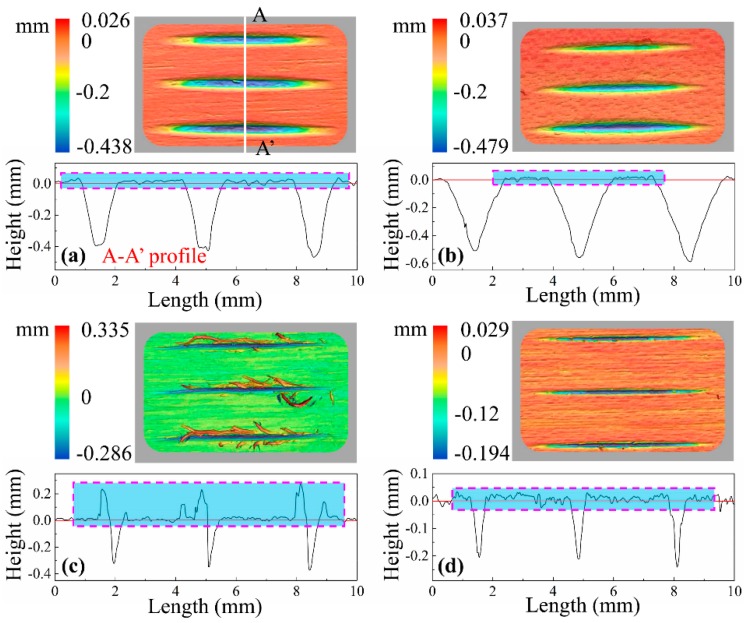
3D morphology and 2D cross-sectional profiles of typical scratches (λ = 0°) cut by: (**a**) spherical cone tool with 2α = 84°, (**b**) spherical cone tool with 2α = 108°, (**c**) triangular pyramid tool with one main cutting edge and two side cutting edges, and (**d**) triangular pyramid tool with two main cutting edges. Note: the 2D cross-sectional profiles were extracted from the deepest point of the scratch like the A-A’ profile.

Figure 4 shows typical 3D morphology of scratches as well as 2D cross-sectional profiles when λ = 0°. There was no clear material pile-up (like raised ridges) on bilateral sides of the scratches cut by spherical cone tools. Meanwhile, the profiles of the scratching grooves generally conformed with the geometry of a spherical cone tool. Looking at the scratches cut by triangular pyramid tools, the basic topographic features are different. In the case of the triangular pyramid tool with one main cutting edge and two side cutting edges, a large amount of incompletely severed fibers projected out over bilateral sides of the scratches. However, the surface quality was much better when the tool had two main cutting edges than when cut by the tool with one main cutting edge and two side cutting edges, and slightly worse than when cut by spherical cone tools.

**Figure 5:**
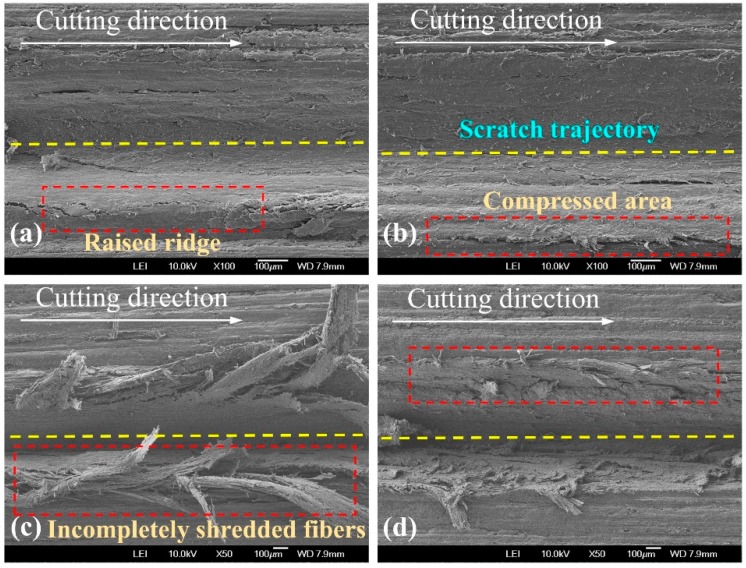
SEM (scanning electron microscope) micrographs of typical scratches (λ = 0°) cut by: (**a**) spherical cone tool with 2α = 84°, (**b**) spherical cone tool with 2α = 108°, (**c**) triangular pyramid tool with one main cutting edge and two side cutting edges, and (**d**) triangular pyramid tool with two main cutting edges.

To further understand how the machining and cutting conditions of the spherical cone tool and triangular pyramid tool affect material removal and surface creation after scratching, SEM micrographs were used as a kind of visual assessment (see Figure 5). Under the four circumstances, the SEM examination of typical scratches exhibited significant fibrillation in the grit-workpiece contact region, which is well conformed with the results of previous studies that negative rake angles produce more micro fibrillating (type III chips) [3,19]. Moreover, when comparing the scratches for spherical cone tools, it was clear that the greater the absolute value of the negative rake angle was, the more micro fibrillation occurred. Only slight raised ridges occurred at both sides of the scratch cut by the spherical cone tool with 2α = 84°. When it comes to 2α = 108°, no clear raised ridges were found, and the critical position of the scratch trajectory and the uncut surface seems to be a highly compressed area due to a bigger rake angle (absolute value). According to a previous study, the effect shows that the material plastic deformation was partially absorbed by the wood anatomical cavities [7]. Due to the existence of sharp cutting edges, the shredding of wood fibers was much easier to observe for scratches cut by triangular pyramid tools. Specifically, a bunch of the incompletely severed fibers and tissues were bent below or above the surface following the movement direction of the single grit, especially for the tool with one main cutting edge and two side cutting edges. To sum up, the cutting geometries of the spherical cone tools induce superficial crushing and fibrillation when scratching along the wood grain in which the results agree with those of other research works [20,21]. In addition, fiber bundles are easier to cut by triangular pyramid tools.

**Figure 6:**
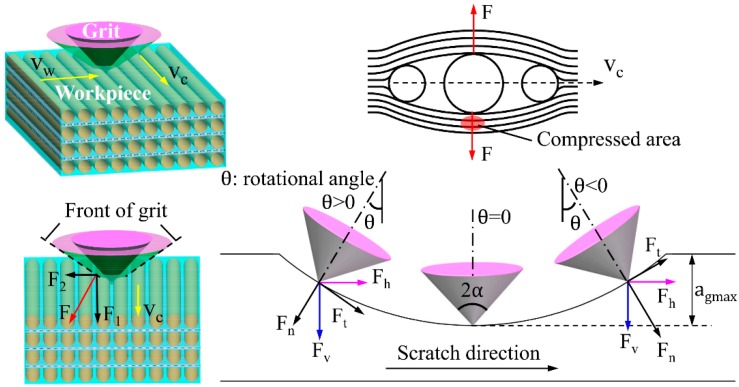
Schematic diagram showing the cutting mechanism for spherical cone tools when λ = 0°.

As shown in Figure 6, the negative rake angle makes the grit strongly inclined to the wood surface, especially during the rubbing stage. The spherical cone grits with tips of a certain curvature radius scratch workpieces in an arch trajectory. From the force analysis of a grit-workpiece interaction, the instant tangential cutting force $F_{t}$ and normal cutting force $F_{n}$ can be separated into a vertical cutting force $F_{v}$ and a horizontal cutting force $F_{h}$, which can be given as:

(4) $$F_{v}\;=\;F_{n}\cos\theta +F_{t}\sin\theta$$

(5) $$F_{h}\;=\;F_{t}\cos\theta -F_{n}\sin\theta$$

where θ is the rotational angle, as illustrated in Figure 6.

Both $F_{h}$ and $F_{v}$ can induce material displacement like elastic and plastic deformation. $F_{v}$ primarily leads to material elastic and plastic deformation, and increases as single grit penetrates deeper into the wood surface, while $F_{h}$ mainly pushes the removed chips forward. According to previous research [7], the subsurface layer is compressed and densified by the $F_{v}$, and, when the plastic deformation exceeds the maximum capability of anatomical cavities absorption, some of the deformed material will be pushed aside, which leads to pile-up ridges caused by a ploughing effect. At the initial stage, most of $F_{n}$ is transformed into material compression. The θ reduces to zero as single grit moves to the deepest point of scratch where $F_{v}$ closes to $F_{n}$, and the force of friction between wood material and the grit conical surface reaches the maximum. Part of the frictional force converts into frictional heat. With the function of other parts of frictional force, the material that cannot be normally compressed is pushed aside, deformed, and flowed away perpendicularly to the conical generatrix. Looking at the front of the grit, the cutting force *F* could be divided into a vertical component $F_{1}$ and a horizontal component $F_{2}$, which can be described as:

(6) $$F_{1}\;=\;F\sin\alpha$$

(7) $$F_{2}\;=\;F\cos\alpha$$

where α is the half included angle of spherical cone tools. According to Equations (6) and (7), $F_{1}$ is larger and $F_{2}$ is smaller for spherical cone grit with 2α = 108. Therefore, more material was compressed by the grit tips. This is the main reason why there is less pile-up ridges at bilateral sides of the scratch trajectory.

**Figure 7:**
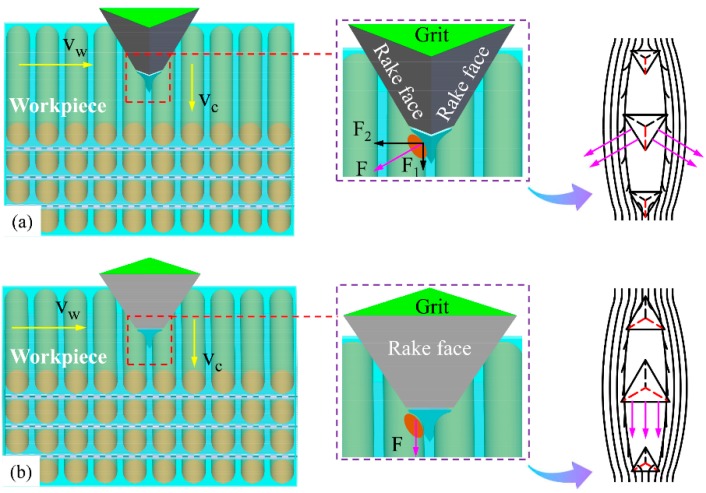
Schematic diagram showing the cutting mechanism for triangular pyramid tools when λ = 0°: (**a**) tool with one main cutting edge and two side cutting edges, and (**b**) tool with two main cutting edges.

Looking at the situation when single grit scratches along the wood grain with one main cutting edge and two side cutting edges, as shown in Figure 7a, the fibers’ mechanical deformation and rupture caused by the main cutting edge proceeds toward the side cutting edges. The sharp main cutting edge could easily cut the fibers into two parts as the single grit penetrates deeper into the wood surface, which leaves the one-end shredded fibers accumulating in front of the rake faces, and the side cutting edges cut the retained fibers thereafter. Consequently, the continuous chip formation appears in the longitudinal fiber cutting. On the one hand, the cutting ability of side cutting edges is relatively weaker. On the other hand, the compressed material partially rebound due to the horizontal component $F_{2}$ of the cutting force *F*. Therefore, clusters of incompletely severed fiber tissues stand above the wood surface after grit passes. However, the grit-workpiece interaction is very different when there are just two main cutting edges. As shown in Figure 7b, stress is more likely to be concentrated in the material ahead of the two main cutting edges, and the material is partially compressed until it is cut by the two main cutting edges nearly simultaneously. Then the shredded material begins to heap up in front of the rake face and flow away with successive cutting force *F* functioning, which is perpendicular to the rake face, and creates better surface quality. Combined with the analysis of SEM micrographs, it can be inferred that the coordinated cutting performance of the two main cutting edges is better than that of the one main cutting edge and two side cutting edges.

#### 3.1.2. Scratching across the Wood Grain (λ = 90°)

**Figure 8:**
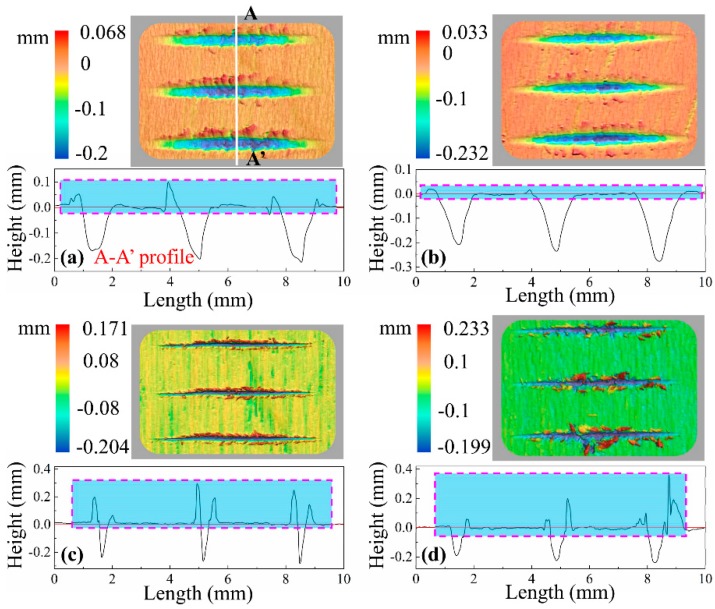
3D morphology and 2D cross-sectional profiles of typical scratches (λ = 90°) cut by: (**a**) spherical cone tool with 2α = 84°, (**b**) spherical cone tool with 2α = 108°, (**c**) triangular pyramid tool with one main cutting edge and two side cutting edges, and (**d**) triangular pyramid tool with two main cutting edges. Note: the 2D cross-sectional profiles were extracted from the deepest point of the scratch like the A-A’ profile.

Figure 8 shows typical 3D morphology of scratches and 2D cross-sectional profiles when λ = 90°, from which it can be found that a large quantity of residual fractured fiber tissues was located at bilateral sides of the scratching grooves except in the case of the spherical cone tool with 2α = 108°, and the triangular pyramid tool with one main cutting edge and two side cutting edges seemed to create more residual fibers bulged on the scratched surface. Basically, profiles of the grooves resemble corresponding cutting geometries.

**Figure 9:**
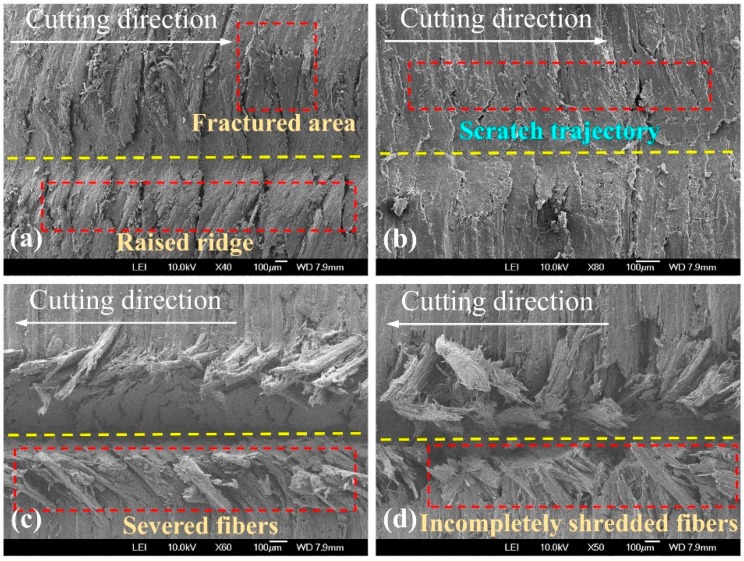
SEM micrographs of typical scratches (λ = 90°) cut by: (**a**) spherical cone tool with 2α = 84°, (**b**) spherical cone tool with 2α = 108°, (**c**) triangular pyramid tool with one main cutting edge and two side cutting edges, and (**d**) triangular pyramid tool with two main cutting edges.

SEM micrographs of typical scratches are shown in Figure 9. Generally, the surface quality of scratches cut by spherical cone tools is better. Nevertheless, numerous crushed wood fibers along the scratch trajectory cut by spherical cone tools indicated that the effective material removal of spherical cone tools is less than that of triangular pyramid tools. Due to the larger angle of 2α = 108°, the ruptured fibers were difficult to rebound. Therefore, the bulged fibers were less clear. In terms of triangular pyramid tools, the surface quality was much worse, which left clusters of partially severed fibers at both sides of the grooves. For a tool with two main cutting edges, the incompletely shredded fibers nearly plugged the groove.

**Figure 10:**
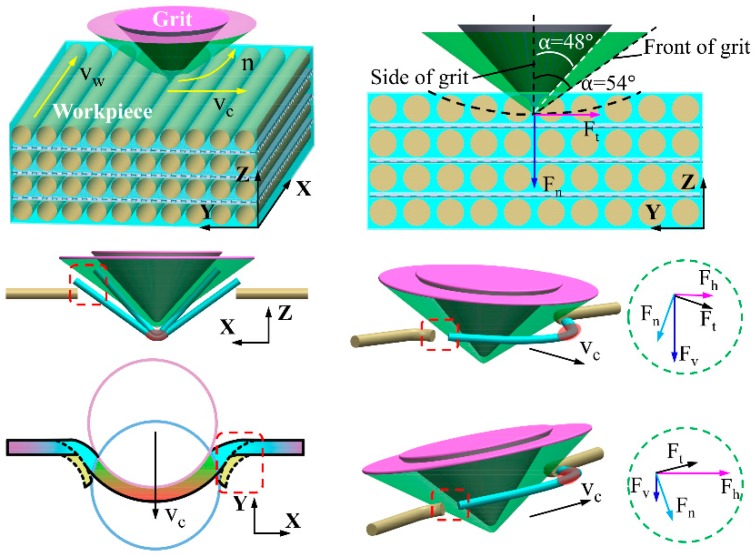
Schematic diagram showing the cutting mechanism for spherical cone tools when λ = 90°.

Based on the above analysis of surface creation, Figure 10 depicts a physical model to describe the cutting mechanism for spherical cone tools when λ = 90°. At the rubbing stage, the rake face of the spherical cone grit is strongly inclined to the workpiece surface. As the grit penetrates deeper, the material deformation could be divided into two situations. For low-density wood species, like Sugar maple, wood fibers are prone to compression underneath the initial surface due to its high porosity. When the resultant of cutting force in the vertical direction $F_{v}$ exceeds the material compressive strength, wood fibers are significantly crushed, which makes two ends of the fractured fibers bulge on the surface. The less the included angle is, the more bulged fibers occur. For high-density wood species, the absorbing capability of anatomical cavities for plastic deformation weakens. Therefore, most wood tissues are torn out as the resultant of cutting force in a horizontal direction $F_{h}$ exceeds the material tensile strength with the chip accumulating in front of the grit and flowing aside.

**Figure 11:**
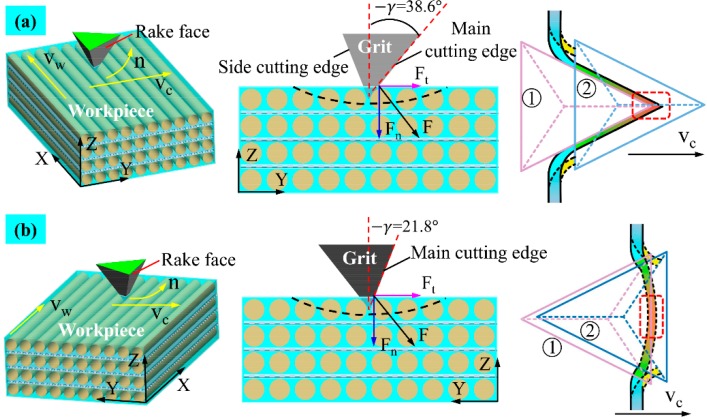
Schematic diagram showing cutting mechanism for triangular pyramid tools when λ = 90°. (**a**) Tool with one main cutting edge and two side cutting edges. (**b**) Tool with two main cutting edges.

The cutting mechanism for triangular pyramid tools when λ = 90° was illustrated in Figure 11. Considering the tool with one main cutting edge and two side cutting edges, the wood fibers are severed at the stress concentration point first by the sharp main cutting edge, as shown in Figure 11a, and, thereafter, the side cutting edges continuously cut the rebounded fibers as the grit moves ahead. Due to the worse cutting ability of the side cutting edges, the one-end severed fiber bundles the bulge on the scratched surface, which is called fibrous stubble. Talking about the situation of two main cutting edges, as shown in Figure 11b, the stress concentration area in front of the rake face is larger, and the materials are severed by the two cutting edges until it reaches ultimate material rupture strength. Meanwhile, a residual part of the severed fibers rebound to its original position and presents fibrous stubble.

### 3.2. Analysis of Cutting Force and Energy Consumption

**Figure 12:**
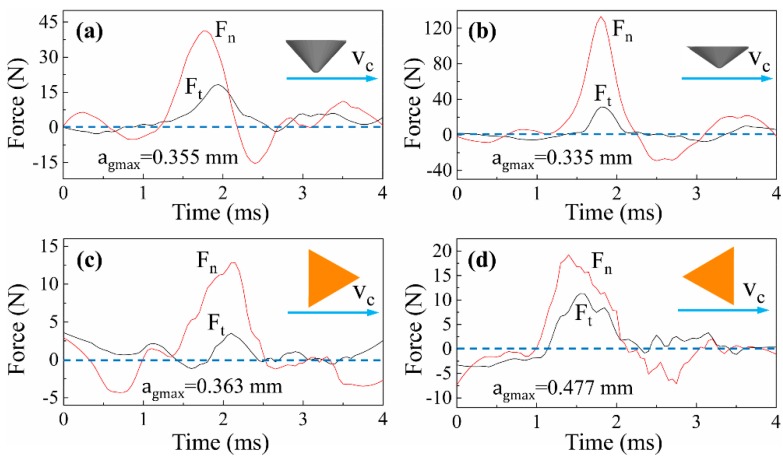
Typical cutting force curve of the scratches (λ = 0°) cut by: (**a**) spherical cone tool with 2α = 84°, (**b**) spherical cone tool with 2α = 108°, (**c**) triangular pyramid tool with one main cutting edge and two side cutting edges, and (**d**) triangular pyramid tool with two main cutting edges.

**Figure 13:**
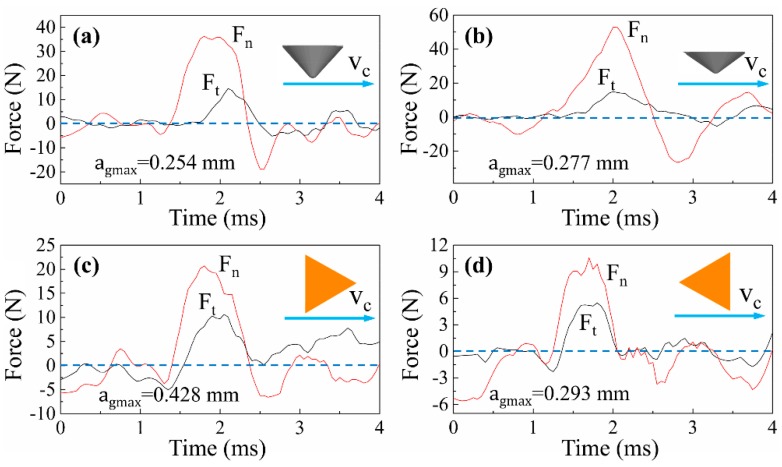
Typical cutting force curve of the scratches (λ = 90°) cut by: (**a**) spherical cone tool with 2α = 84°, (**b**) spherical cone tool with 2α = 108°, (**c**) triangular pyramid tool with one main cutting edge and two side cutting edges, and (**d**) triangular pyramid tool with two main cutting edges.

Figure 12 and Figure 13 show the typical signals of cutting forces when scratching along and across the wood grain, respectively. It was found that the normal force $F_{n}$ was strikingly larger than tangential force $F_{t}$ in all cases, which was a common and typical feature in cutting with negative rake angles. The cutting forces of all cases increased until the grit penetrates to the deepest point of the scratch trajectory, and then decreased monotonically. In addition, the cutting force curves of triangular pyramid tools present more oscillation than those of spherical cone tools for both λ = 0° and λ = 90°. The difference indicated that the cutting forces could be affected by the tool geometries, specifically the sharp cutting edges. Compared with the cutting force curves of λ = 0° and λ = 90°, it was observed that the curves of λ = 0° were relatively smoother than that of λ = 90°. When cutting along the wood grain, the wood fibers are mostly crushed and shredded. However, the wood fibers are prone to be severed or fractured by tensile failure when cutting across the wood grain. The transverse fibers show huge heterogeneity and the critical condition for fiber rupture is diverse, as well as the force loss and loading in the instance of rupture. Hence, the cutting process behaves more complexly as grit moves perpendicularly to the wood fibers.

In the process of sanding wood materials, the tangential sanding force increases as normal sanding force increases, as along with the sanding depth in a certain range. Other studies found that the sanding efficiency decreased with the decrease of tangential force under a constant normal force [22]. In this study, the cutting force ratio defined as *F_t_*/*F_n_* was used to evaluate the cutting efficiency. An increase in the ratio suggests that more cutting occurs due to an increase in the tangential force relative to the normal force. Looking at the energy utilization, the overall cutting power means the specific energy consumed by tangential force *F_t_* in a unit time, which can be applied to assess the energy consumption in the cutting process. 

**Figure 14:**
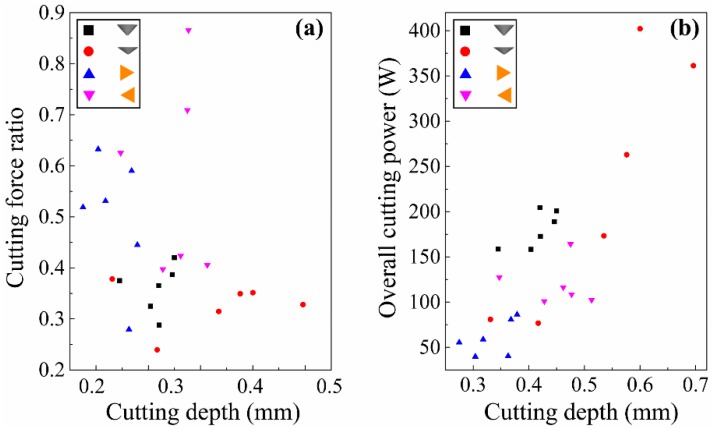
Analysis of cutting efficiency and energy consumption (λ = 0°): (**a**) experimental cutting force ratios for single grits and (**b**) overall cutting power for single grits.

As Figure 14a shows, triangular pyramid tools were performing more cutting than the spherical cone tools when λ = 0°, which strongly manifests that the tools with clear cutting edges show better cutting ability in this study. Importantly, it was found that the overall cutting power of spherical cone tools was significantly higher than that of triangular pyramid tools from Figure 14b, which conforms with the above analysis of material removal and surface creation. During sanding or a grinding process, the material removal mechanism is commonly described in three phases, which are rubbing, ploughing, and cutting [23]. According to previous research, plastic deformation presents pile-up and surface densification for wood materials [7]. Specifically, the spherical cone tools have poor cutting geometries, and, therefore, the material is not efficiently cut. Instead, more energy is used to make plastic material deformation, which presents pile-up ridges at bilateral sides of the scratches (see Figure 5), as well as plastic surface densification. When spherical cone tools with different included angles were compared, it was noted that tools with 2α = 84° presented higher cutting efficiency than 2α = 108°. Moreover, tools with 2α = 108° consumed more energy relatively, which could be due to the size effect. It is commonly believed that the bluntness of tool contributes greatly to the size effect, because the tool merely presses on and rubs against the surface without cutting since the tool edge has been rounded [24,25].

**Figure 15:**
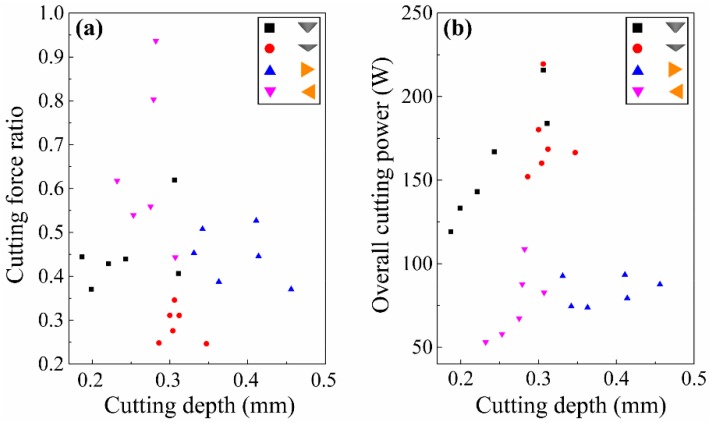
Analysis of cutting efficiency and energy consumption (λ = 90°): (**a**) experimental cutting force ratios for single grits, and (**b**) overall cutting power for single grits.

Looking at the situation when λ = 90°, the results are basically the same as λ = 0°. From Figure 15, it can be shown that the cutting force ratio of spherical cone tools were apparently less than that of triangular pyramid tools. Meanwhile, the overall cutting power for spherical cone tools was remarkably higher than that for triangular pyramid tools. When scratching across wood grains, wood fibers are mainly torn out by tensile failure for Sugar maple workpieces, and tools with sharp cutting edges make it easier to remove chips. Combined with the SEM images of scratches in Figure 9, it can be inferred that the cutting ability of spherical cone tools are much worse than tools with sharp cutting edges. Extra energy is consumed to make material plastic deformation. Furthermore, wood fibers are vastly compressed and crushed.

## 4. Conclusions

To investigate the cutting characteristics of dull abrasive grits and grits with a well-defined cutting edge for wood materials, spherical cone tools and triangular pyramid tools were used in this study and a series of single-grit scratching tests, with two main cutting directions (along and across the wood grain). The conclusions can be drawn as follows.

(1) Physical cutting models for each case were established to interpret material deformation and surface creation. Sharp cutting edges yield stress concentration of the workpiece much more easily than the conical surface, which makes it more efficient to sever wood fibers.
(2) The plastic pile-up was not clear when scratching along the wood grain and one-end severed fiber bundles tended to bulge on the surface like fibrous stubble when scratching across the wood grain.
(3) A new method was developed to evaluate the overall cutting power. For scratching along and across the wood grain, the cutting force ratio of spherical cone tools were apparently less than that of triangular pyramid tools. Meanwhile, the overall cutting power for spherical cone tools was remarkably higher than that for triangular pyramid tools. It indicates that cutting performance takes a larger proportion for triangular pyramid tools, while vast energy is consumed to make material plastic deformation for spherical cone tools.
